# Building a Point of Care Ultrasound (POCUS) Curriculum in Undergraduate Medical Education Through Stepwise Development and Assessment

**DOI:** 10.24908/pocusj.v10i01.18116

**Published:** 2025-04-15

**Authors:** Nancy L. Hagood, Romik Srivastava, Marc E. Heincelman, Meghan K. Thomas

**Affiliations:** 1 Division of Hospital Medicine, Department of Medicine, Medical University of South Carolina, Charleston, SC, USA; 2 Division of Gastroenterology, Department of Medicine, Medical University of South Carolina, Charleston, SC, USA

**Keywords:** point of care ultrasound, POCUS, curriculum development, undergraduate medical education, clerkship, internal medicine

## Abstract

**Background:**

Point of care ultrasound (POCUS) training is increasingly incorporated in undergraduate medical education (UME). However, limited resources and lack of standard guidelines lead to questions regarding the most effective curriculum and assessment method. The authors aimed to develop a longitudinal UME POCUS curriculum through staged intervention. Year 1, which involved simulation alone, led to improved confidence without adequate knowledge. The authors hypothesized that the addition of resident-led workshops alongside faculty-led lectures would improve POCUS knowledge and confidence among third-year medical students.

**Methods:**

A prospective cohort study of third-year students on the Internal Medicine (IM) clerkship at a large academic medical center was performed, assessing efficacy of stepwise POCUS curriculum development. Previously implemented year 1 involved comparing the control cohort receiving baseline POCUS education on rounds with the experimental cohort that had access to a high-fidelity POCUS simulator. The year 2 cohort added hands-on resident-led POCUS workshops. The year 3 cohort added faculty-led lectures. All cohorts completed pre- and post-intervention confidence and knowledge-based examinations. The year 1 control cohort served as a control for the current study.

**Results:**

A total of 69 and 102 students completed both pre-/post-tests among year 2 and 3 cohorts, respectively. Both cohorts demonstrated statistically significant improvement in POCUS knowledge and confidence, with greater magnitude of improvement in year 3 with overall knowledge improving from 49.9% to 66.7% on pre- to post-intervention examination (p<0.0001).

**Conclusion:**

While simulation alone was insufficient to instill knowledge, the addition of resident-led workshops and faculty-led lectures demonstrated benefits in POCUS knowledge and confidence among medical students and represents a sustainable model of training.

## Background

Point of care ultrasound (POCUS) is a “goal-directed, bedside exam performed by a healthcare provider in real-time to answer a specific diagnostic question or guide an invasive procedure [1].” Use of POCUS has expanded rapidly in recent years due to advances in machine portability and quality, adoption of training curriculums, and extensive literature describing improvements in diagnostic expediency, procedural safety, and patient outcomes [1–3]. In addition to its clinical applications, POCUS is increasingly used as an adjunctive educational tool for diagnostic and physical exam skills, better understanding of anatomic relationships and physiologic concepts, and safer procedural guidance [3,4].

Given its widespread use and demonstrated benefits, the Accreditation Council for Graduate Medical Education (ACGME) has named POCUS a major competency in Emergency Medicine, Anesthesiology, and Family Medicine [5–7]. Additional Graduate Medical Education (GME) residency programs, including Internal Medicine (IM), Surgery, and Obstetrics and Gynecology, have incorporated POCUS education into their standard curricula [8–10]. The addition of POCUS at the GME level has encouraged Undergraduate Medical Education (UME) programs to incorporate ultrasonography training into their educational mission and structure to promote early exposure to POCUS [3,4,11]. POCUS is a user-dependent technical skill, and early exposure in UME can promote competency [3]. Additionally, there is strong interest and enthusiasm for POCUS training among medical students [12].

While 79% of medical schools believe that POCUS training should be incorporated in UME curriculum, only 62% have done so [4]. Curricular design and delivery is variable with no national standards or guidelines [3]. Those seeking to implement a POCUS curriculum face common barriers of insufficient curricular time, paucity of trained faculty, and lack of financial support for purchase and maintenance of POCUS equipment dedicated to educational use [4,13].

Our medical school, like other institutions, is developing a longitudinal, multi-year UME POCUS curricula. Here, we present the results of years 2 and 3 of a stepwise intervention to develop a longitudinal POCUS curriculum during the 6-week IM clerkship at a large academic medical center in the Southeastern United States. In year 1 of curriculum development, we demonstrated that use of high-fidelity simulation alone increased POCUS confidence among medical students. However, knowledge scores were not as robust as expected, suggesting an undesirable Dunning-Kruger effect or cognitive bias [14]. We therefore implemented additional educational modalities in years 2 and 3 of curriculum development with the goal of educationally significant knowledge improvement. We hypothesized that implementation of hands-on resident-led POCUS workshops in year 2 and longitudinal faculty-led POCUS lectures in year 3 would further improve both POCUS confidence and knowledge among third-year medical students.

## Methods

A prospective cohort study was performed assessing the efficacy of a POCUS curriculum implemented during the 6-week IM clerkship at the Medical University of South Carolina (MUSC). Student participation in the study survey and POCUS simulation was voluntary; however, the curriculum itself comprised of the workshop and lectures was a required expectation of all clerkship students. The MUSC Institutional Review Board reviewed the study and deemed it exempt. The POCUS curriculum was developed, implemented, and assessed in stepwise fashion over a total of three years.

Year 1 was implemented from January 2021 through May 2021. The year 1 control cohort consisted of students who received only informal, or “baseline,” POCUS education provided by faculty and residents during teaching rounds. The year 1 experimental cohort consisted of students who, in addition to “baseline” POCUS education during teaching rounds, had access to a high-fidelity POCUS simulator for two 5-hour self-directed sessions per week during the clerkship. These students were referred to as the “simulation cohort.” Available simulation modules included POCUS basic skills, cardiac POCUS, COVID-19, and lung POCUS. Each module included didactic videos and algorithms, followed by hands-on practice cases with the simulator providing real-time feedback and probe position guidance [14]. Results from year 1 intervention were previously published [14], and the year 1 control cohort was used again as a control in the current study. These students were referred to as the “control cohort.”

Year 2 was implemented from July 2021 through June 2022 and involved the implementation of a 3-hour hands-on resident-led POCUS workshop for all students during the IM clerkship. These students were referred to as the “workshop cohort.” The workshop was held from 5-8 PM, and IM residents served as both teachers and volunteer standardized patients for the workshop. All residents were actively enrolled in a longitudinal POCUS curriculum as part of their GME didactic training. The ratio of teaching residents to students was 1:3-4. The workshop included an introduction to POCUS and then focused on high-yield POCUS exams, including lung, cardiac, focused assessment with sonography in trauma (FAST), and deep venous thrombosis (DVT) exams. Additionally, all students had access to a high-fidelity POCUS simulator for independent, self-directed use throughout the clerkship based on year 1 intervention.

Year 3 was implemented from July 2022 through June 2023 and involved the addition of longitudinal faculty-led POCUS lectures for all students throughout the 6-week clerkship. These students were referred to as the “lecture cohort.” A total of four lectures were given by a single faculty member throughout the clerkship. Lecture topics included introduction to POCUS, lung POCUS, cardiac POCUS, and abdominal and DVT POCUS. The resident-led workshops were maintained but were moved to 2-5 PM, and standardized patients were hired so that residents served as teachers only. Students continued to have access to a high-fidelity POCUS simulator for independent, self-directed use throughout the clerkship.

A pre- and post-intervention survey was completed at the beginning and end of the clerkship during all stages of intervention. The pre-intervention survey included demographic and needs assessment questions. Both the pre- and post-intervention surveys included confidence and knowledge-based examinations that reviewed ultrasound physics, procedural use, image acquisition, recognition, and interpretation. The same survey was used for all cohorts. The survey was previously validated [15] and is included in the supplementary material. Students' confidence and perception of POCUS value was assessed using a 5-point Likert scale ranging from “1=not at all” to “5=extremely.” Paired t-tests were performed to compare pre- and post-intervention Likert scale responses and mean scores knowledge-based categorical questions. The data were analyzed using SAS version 9.4 (SAS Institute Inc., Cary, NC).

## Results

Among clerkship students included in the study, a total of 44% (69/156) and 63% (102/161) completed both the pre- and post-intervention surveys among the workshop and lecture cohorts, respectively. Of students in the workshop cohort, approximately two-thirds (62%, 43/69) reported no prior POCUS training and nearly all (93%, 64/69) reported rarely or never using POCUS in their current practice. Similarly, among students in the lecture cohort, 73% (74/102) reported no prior POCUS training and 93% (95/102) reported rarely or never using POCUS in their current practice (Table 1).

**Table 1.** Pre-intervention demographics among the workshop and lecture cohorts.

	Workshop Cohort Frequency in Percentage (N=69)	Lecture Cohort Frequency in Percentage (N=102)
**Prior POCUS training**		
Yes	38% (26)	28% (28)
No	62% (43)	73% (74)
**Frequency of current POCUS use**		
Never	51% (35)	50% (51)
Rarely	42% (29)	43% (44)
Once per month	3% (2)	3% (3)
Once per week	4% (3)	4% (4)
Daily	0% (0)	0% (0)

**Figure 1.**
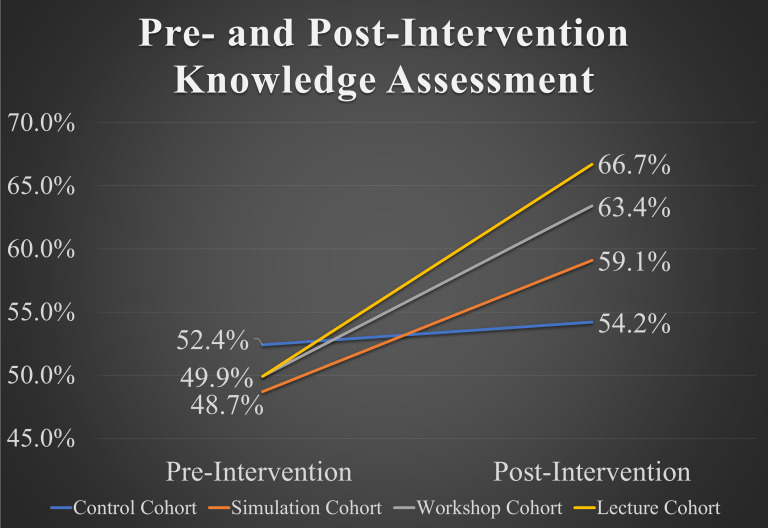
Pre- and post-intervention examination results for overall POCUS knowledge.

With regard to POCUS knowledge, the workshop and lecture cohorts demonstrated statistically significant improvement in overall POCUS knowledge. There was a greater magnitude of improvement among the lecture cohort as overall POCUS knowledge improved from 49.9% to 66.7% on pre- to post-intervention examination, respectively (p<0.0001) (Table 2, Figure 1). Both cohorts also displayed statistically significant improvement in the individual knowledge-based domains of physics, image recognition, and image interpretation (Table 2).

**Table 2.** Pre- and post-intervention POCUS knowledge examination results among the workshop and lecture cohorts.

	**Workshop Cohort (N=69)**		
	Pre- Intervention Mean % Correct	Post- Intervention Mean % Correct	P*
Total Knowledge Score	49.9%	63.4%	**<0.001**
Physics	53.8%	63.2%	**0.0010**
Procedural Use	52.9%	60.1%	0.0953
Image Acquisition	32.4%	42.5%	**0.0112**
Image Recognition	52.3%	69.3%	**<0.0001**
Image Interpretation	39.3%	58.4%	**<0.0001**
	**Lecture Cohort (N=102)**		
	Pre-Intervention Mean % Correct	Post-Intervention Mean % Correct	P**
Total Knowledge Score	49.9%	66.7%	**<0.0001**
Physics	52.7%	67.7%	**<0.0001**
Procedural Use	61.3%	63.0%	0.5399
Image Acquisition	30.3%	35.3%	0.1407
Image Recognition	46.6%	71.4%	**<0.0001**
Image Interpretation	40.6%	67.0%	**<0.0001**

N.B. P-values with bolded font designate those <0.05;

P* value between pre- and post-intervention workshop cohort;

P** value between pre- and post-intervention lecture cohort.

Regarding POCUS confidence, the workshop and lecture cohorts each demonstrated statistically significant improvement in POCUS confidence on pre- to post-intervention analysis with the following questions: “How confident are you in your knowledge of POCUS?”; “How confident do you feel performing procedures?”; and “How confident are you in performing procedures with the guidance of POCUS?” (Table 3).

**Table 3.** Pre- and post-intervention POCUS confidence survey results among the workshop and lecture cohorts.

	Workshop Cohort (N=69)				Lecture Cohort (N=102)			
Question	Pre- Intervention Mean (SD)	Post- Intervention Mean (SD)	Pre-/Post-Intervention Difference	P*	Pre-Intervention Mean (SD)	Post-Intervention Mean (SD)	Pre-/Post-Intervention Difference	P**
How confident are you in your knowledge of POCUS?	1.94 (0.75)	2.81 (0.73)	+0.86	**<0.0001**	1.85 (0.74)	3.13 (0.73)	+1.28	**<0.0001**
How confident do you feel performing procedures?	1.70 (0.86)	2.43 (0.92)	+0.76	**<0.0001**	1.46 (0.70)	2.61 (0.91)	+1.15	**<0.0001**
How confident are you in performing procedures with the guidance of POCUS?	1.51 (0.68)	2.41 (1.05)	+0.90	**<0.0001**	1.39 (0.60)	2.65 (0.89)	+1.26	**<0.0001**
How beneficial do you feel POCUS skills will be for the health of your patient?	4.33 (0.92)	4.48 (0.78)	+0.14	0.3191	4.07 (1.01)	4.28 (0.81)	+0.21	0.0941
How beneficial do you feel POCUS can be in aiding your diagnostic evaluation and reasoning?	4.51 (0.78)	4.46 (0.78)	−0.05	0.7434	4.21 (0.94)	4.26 (0.87)	+0.05	0.6848
How beneficial do you feel that POCUS simulation training will be to your future medical career?	4.45 (0.83)	4.33 (0.97)	−0.13	0.4511	4.24 (0.97)	4.22 (0.83)	−0.02	0.8765
How likely are you to incorporate POCUS into your practice?	4.25 (0.90)	4.30 (0.95)	+0.05	0.6453	4.03 (1.04)	4.17 (0.93)	+0.14	0.3122
How likely are you to use POCUS as an aid in urgent or emergent patient management?	4.26 (0.85)	4.32 (0.88)	+0.05	0.6952	4.01 (1.02)	4.12 (0.93)	+0.11	0.4300
POCUS is a general procedure a physician should be competent in.	1.74 (1.17)	1.39 (0.71)	−0.35	0.0369	1.53 (0.91)	1.73 (1.12)	+0.20	0.1710
POCUS can help in the recognition of a patient requiring urgent or emergent care and better allow the clinician to initiate evaluation and management.	1.67 (1.08)	1.49 (0.92)	−0.17	0.3099	1.53 (0.88)	1.67 (1.00)	+0.14	0.2978

N.B. Variables measured using 5-point Likert scale ranging from “1=not at all” to “5=extremely”; P-values with bolded font designate those <0.05;

P* value between pre- and post-intervention workshop cohort;

P** value between pre- and post-intervention lecture cohort.

## Discussion

Implementation of years 2 and 3 of a multi-stage intervention to develop a longitudinal POCUS curriculum during the IM clerkship demonstrated that hands-on resident-led POCUS workshops increased POCUS knowledge and confidence among medical students, and subsequent addition of longitudinal faculty-led POCUS lectures further improved POCUS knowledge. Advantages of this stepwise, multimodal curricular approach include increased hands-on skill development and decreased need for trained faculty by utilizing residents as teachers.

Paucity of trained faculty is a common barrier to implementing POCUS training programs, and trained faculty who do exist are often prioritized to train faculty and residents before students. Our study shows successful POCUS training programs are feasible even with limited resources, as a few dedicated faculty members can train residents [15] who can then assist with educating students. Faculty development requires time and investment. However, it is essential to build capacity [3], and our results demonstrate that even limited faculty can implement effective curricula through longitudinal lecture series and by cultivating residents as teachers.

Notably, POCUS knowledge scores improved pre- to post-intervention in both the workshop and lecture cohorts but not equally and not in all knowledge domains. Unsurprisingly, procedural use and image acquisition demonstrated little to no statistically significant improvement, suggesting that more clinical and hands-on experience is required to sufficiently instill these skills. On the other hand, image recognition and image interpretation revealed the greatest absolute improvement. This improvement occurred both with and without the addition of faculty, suggesting that these skills may be easier for students to grasp and an easier subject to deliver through traditional teaching methods. These results are valuable when structuring future longitudinal curricula and identifying which areas require educational emphasis and devoted resident/faculty time.

While student confidence improved from pre- to post- intervention survey, there was no change in questions regarding perception of benefits of POCUS and likeliness to use. This lack of change was primarily due to high pre-intervention Likert scores, demonstrating high levels of enthusiasm and engagement even pre-intervention. Students believed POCUS was beneficial and aided patient management, and they believed this even before they had exposure to the curriculum.

A primary limitation of our study was resident time and availability, as workshops depended on resident volunteers. During initial implementation of resident-led workshops in year 2, we faced attention fatigue with workshops at the end of the day, as well as limited availability of POCUS probes dedicated to educational use. After collecting student feedback and outcomes data, we were able to change the time of day of workshops in year 3, hire standardized patients, and purchase portable POCUS probes dedicated to medical student use. The allocation of educational time and financial resources toward POCUS training was facilitated through stepwise evaluation of our curriculum. Additional limitations of our study included variable levels of expertise and education provided by residents during workshops, which cannot be controlled for and may represent a confounding variable given our small sample sizes. As well, student participation in the survey and POCUS simulation components were voluntary, which may underestimate the impact of intervention. Student time spent independently using the simulator also was not measured during this study. Finally, our pre- and post-intervention assessments primarily focused on POCUS perceptions (i.e., confidence and use) and knowledge. Unfortunately, skill level was not specifically assessed in our study; however, residents often performed a skills check-off during the workshops.

There are multiple benefits to implementing curricula through stepwise intervention. First, training faculty requires time and investment, and utilizing alternative educational modalities may be necessary in early stages. Second, educational time is valuable. POCUS is an important skill that should be incorporated within the larger UME framework. Thoughtful design, implementation, and assessment of curricular interventions ensures effectiveness and improvement in learner outcomes [3]. Third, when resources are limited, stepwise assessment allows appropriate allocation of resources. Our results suggest that investment in POCUS-trained educators, standardized patients, and dedicated ultrasound equipment for educational use is prudent for the development of a successful and sustainable POCUS training curriculum.

Finally, while there is much excitement and enthusiasm surrounding POCUS education and use, our results emphasize the importance of evaluating knowledge and skill in conjunction with confidence given the prevalence of cognitive bias in education. Although confidence increased in all three years of intervention, improvement in individual knowledge scores was present only with the addition of workshops and lectures in years 2 and 3, suggesting a Dunning-Kruger effect prevalent particularly in year 1 with use of simulation alone [14]. This type of cognitive bias is particularly important to recognize when teaching a technical skill like POCUS. Perceived confidence, coupled with little knowledge, may result in indiscriminate use of POCUS and patient harm from incidental findings, inaccurate diagnoses, and additional unnecessary testing [2].

## Conclusions

Initial intervention demonstrated that simulation alone is insufficient to instill POCUS knowledge among medical students [14]. Our current curriculum presents a sustainable model of POCUS training and combines hands-on resident-led skills workshops with lectures and simulation to ensure improvements in both knowledge and confidence among medical students. Building upon the success of our current curriculum through detailed evaluation, next steps will include a longitudinal POCUS curriculum that spans the duration of medical school, incorporating gross anatomy, physiology, and clinical skills courses.


