# Supplementary material for: Building a Point of Care Ultrasound (POCUS) Curriculum in Undergraduate Medical Education Through Stepwise Development and Assessment
Source: POCUS J. 2025 Apr 15;10(1):32–7. doi: 10.24908/pocusj.v10i01.18116 (PMC12057471; doi:10.24908/pocusj.v10i01.18116)
Supplement: Supplementary file 1 [file pocusj-10-01-18116-s001.pdf]

Background for Ultrasound Survey

We are reaching out to ask for your participation in a research study being conducted at MUSC to evaluate the efficacy of simulation technology on ultrasound knowledge and use.

The following survey is a tool used to obtain baseline information of students' knowledge and skills of point-of-care ultrasound. We will be providing a written and skills-based pre-test. There will be a similar post-test and retention test to follow. The results of these tests may be used to create and improve a Point-of-Care ultrasound curriculum for clinical students. The purpose of the study is to improve educational curriculum regarding point-of-care ultrasound. Although the intent of the study is research, you may benefit through participation by improving knowledge and skills in ultrasound.

Participation in this survey is voluntary and has no bearing on your Internal Medicine Clerkship grade or evaluation. Your consent will be implied by submission of the survey. All of your answers and personal information, including name and email, will be kept confidential, however, there is a small risk that this information becomes accessible by the study principal investigators. The risk is low since your name and email will be kept confidential and instead, a study ID number will be linked to your responses and simulation scores.

If you have any questions or concerns please contact the investigators, Maggie Thomas at andersmk@musc.edu or Bill McManigle at mcmanigl@musc.edu.

The survey will take about 10-15 minutes to complete. Thank you for your participation.

Ultrasound ID

____
(Obtained during orientation (PDF file))

Training Level (MS#, PGY#)

____

Gender

◯ Male
◯ Female
◯ Other

Please provide identified gender:

____

Prior Ultrasound Training

◯ Yes
◯ No

How often do you use ultrasound in your current practice?

◯ Never
◯ Rarely
◯ Once per month
◯ Once per week
◯ Every day

Ultrasound Simulation Survey

Time

about survey

How confident are you in your knowledge of ultrasound?	◯ Not at all<br>◯ Slightly<br>◯ Moderately<br>◯ Quite<br>◯ Extremely
How confident do you feel performing procedures?	◯ Not at all<br>◯ Slightly<br>◯ Moderately<br>◯ Quite<br>◯ Extremely
How confident are you in performing procedures with the guidance of ultrasound?	◯ Not at all<br>◯ Slightly<br>◯ Moderately<br>◯ Quite<br>◯ Extremely<br>(Confidence with US guided procedures)
How beneficial do you feel ultrasound skills will be for the health of your patient?	◯ Not at all<br>◯ Slightly<br>◯ Moderately<br>◯ Quite<br>◯ Extremely
How beneficial do you feel ultrasound can be in aiding your diagnostic evaluation and reasoning?	◯ Not at all<br>◯ Slightly<br>◯ Moderately<br>◯ Quite<br>◯ Extremely
How beneficial do you feel that ultrasound simulation training will be to your future medical career?	◯ Not at all<br>◯ Slightly<br>◯ Moderately<br>◯ Quite<br>◯ Extremely
How likely are you to incorporate ultrasound into your practice?	◯ Not at all<br>◯ Slightly<br>◯ Moderately<br>◯ Quite<br>◯ Extremely
How likely are you to use ultrasound as an aid in urgent or emergent patient management?	◯ Not at all<br>◯ Slightly<br>◯ Moderately<br>◯ Quite<br>◯ Extremely
Point-of-care ultrasound is a general procedure a physician should be competent in.	◯ Strongly Agree<br>◯ Agree<br>◯ Neutral<br>◯ Disagree<br>◯ Strongly Disagree

Point-of-care ultrasound can help in the recognition of a patient requiring urgent or emergent care and better allow the clinician to initiate evaluation and management.	◯ Strongly Agree<br>◯ Agree<br>◯ Neutral<br>◯ Disagree<br>◯ Strongly Disagree

Describe this probe in questions below.

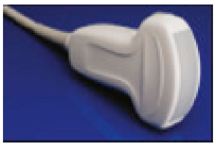

Name of probe (shown above):	__________________________
Wavelength of probe (shown above):	◯ Short ◯ Long
Frequency of probe (shown above):	◯ Low ◯ High
Penetration of probe (shown above):	◯ Shallow ◯ Deep
Image Resolution of probe (shown above):	◯ High ◯ Low

Describe this probe in questions below.

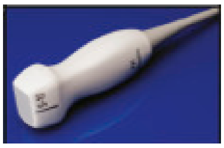

Name of probe (shown above):	__________________________
Wavelength of probe (shown above):	◯ Short ◯ Long
Frequency of probe (shown above):	◯ Low ◯ High
Penetration of probe (shown above):	◯ Shallow ◯ Deep
Image Resolution of probe (shown above):	◯ High ◯ Low

Describe this probe in questions below.

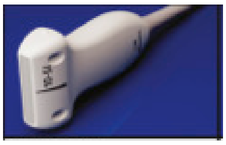

Name of probe (shown above):	__________________________
Wavelength of probe (shown above):	◯ short ◯ long

Frequency of probe (shown above):	◯ Low ◯ High
Penetration of probe (shown above):	◯ Shallow ◯ Deep
Image Resolution of probe (shown above):	◯ High ◯ Low
Name the appropriate probe for central line placement.	◯ Linear<br>◯ Curvilinear<br>◯ Phased array<br>◯ None of the above
Name the appropriate probe for paracentesis.	◯ Linear<br>◯ Curvilinear<br>◯ Phased array<br>◯ None of the above
Name the appropriate probe for thoracentesis.	◯ Linear<br>◯ Curvilinear<br>◯ Phased array<br>◯ None of the above
Ultrasound-guided lumbar punctures require the patient to be lying down.	◯ True<br>◯ False
In doppler mode, the red color always signifies arterial blood.	◯ True<br>◯ False
Increasing gain will yield a brighter image.	◯ True<br>◯ False

Use this picture to answer the question below.

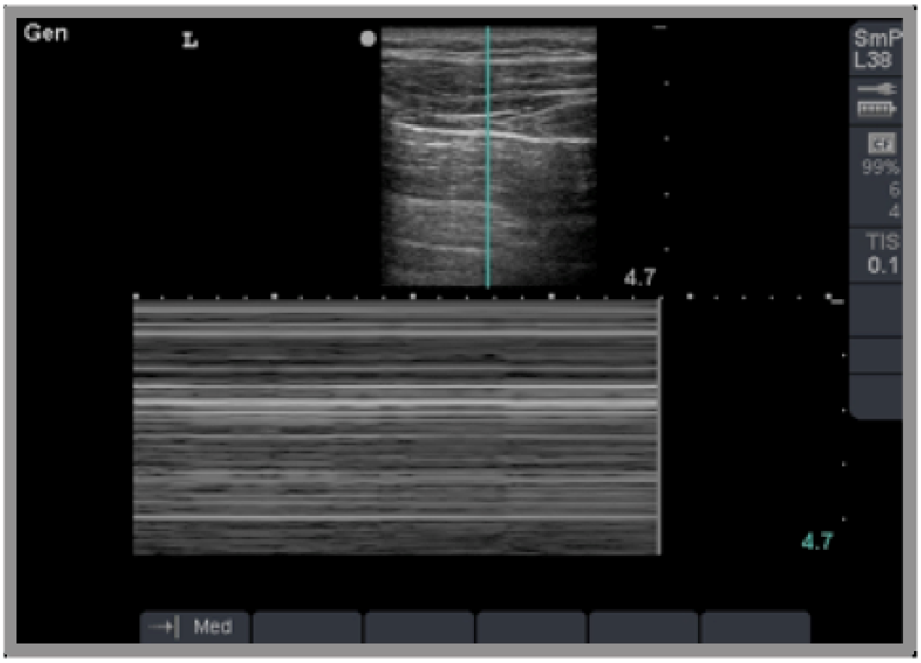

	True	False
Doppler-mode was used to obtain the image seen above.	◯	◯

Please use this image to answer questions below.

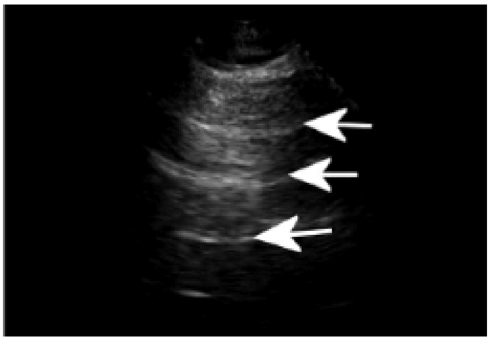

What do the white arrows indicate?

◯ A-lines
◯ B-lines
◯ Consolidation
◯ Z-lines

The image above shows evidence of pulmonary edema.

◯ True
◯ False

Please use this image to answer questions below.

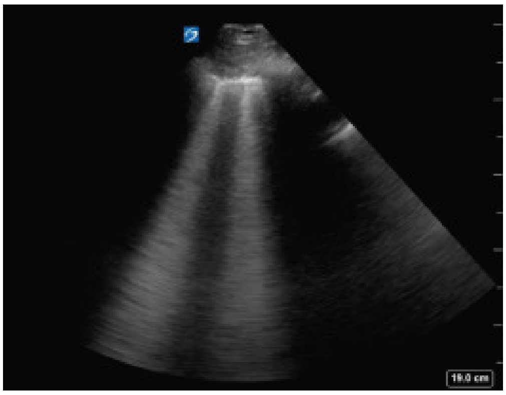

What artifact is depicted in the image above?

◯ A-lines
◯ B-lines
◯ Consolidation
◯ Z-lines

Give an example/diagnosis of what this artifact can indicate.

____
(example of what b-lines can indicate)

Please use this image to answer questions below.

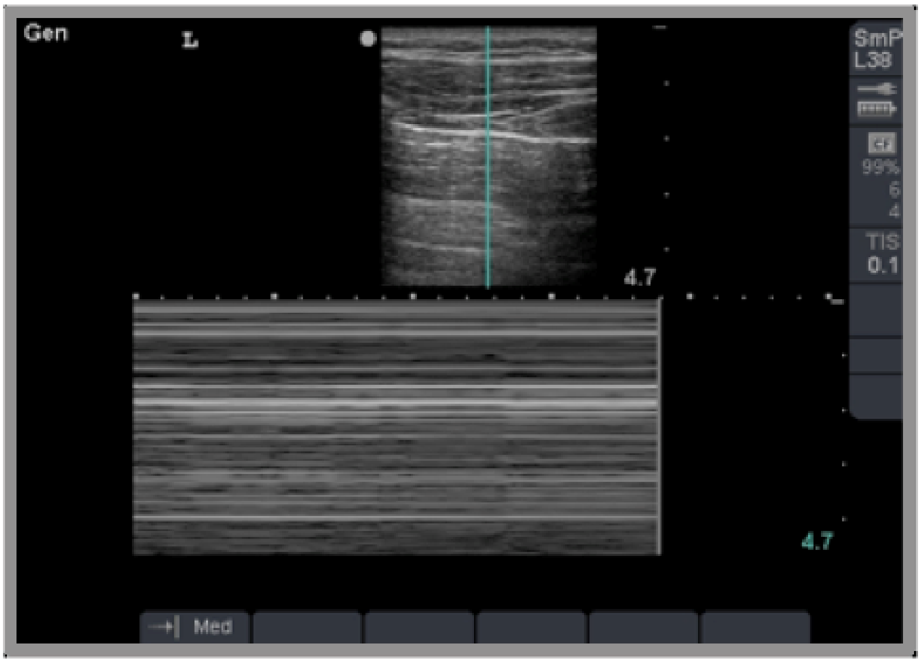

Please name the "sign" that is shown in the picture above. __________________________________

Interpret the meaning of the "sign" shown in the picture above (i.e. what does it mean clinically). __________________________________

Please use this image to answer the questions below.

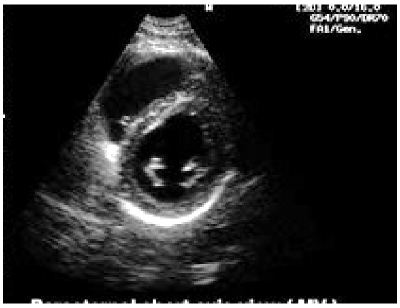

Name the cardiac view depicted above.

- ◯ Parasternal Long
- ◯ Parasternal Short
- ◯ Apical 4-chamber
- ◯ Subcostal
- ◯ Transesophageal
- ◯ None of the above

What two chambers are depicted in the image above.

- ☐ Right atrium
- ☐ Right ventricle
- ☐ Left atrium
- ☐ Left ventricle
- ☐ Carotid sinus
- ☐ Aorta
- ☐ IVC

Please use this image to answer the questions below.

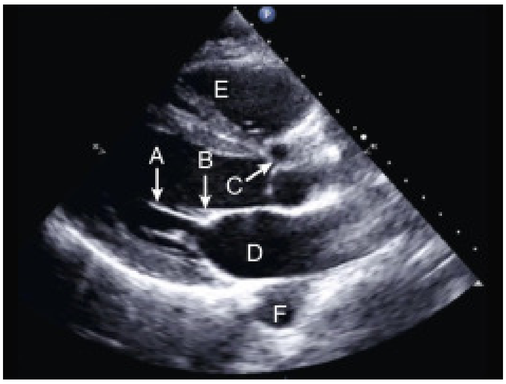

Name the cardiac view depicted above.

◯ Parasternal Long
◯ Parasternal Short
◯ Apical 4-chamber
◯ Subcostal
◯ Transesophageal
◯ None of the above

Please name each of the structures labeled in the image above.

	Mitral valve leaflet	Right Ventricle	Descending aorta	Chordae Tendinae	Left atrium	Aortic valve
A.	◯	◯	◯	◯	◯	◯
B.	◯	◯	◯	◯	◯	◯
C.	◯	◯	◯	◯	◯	◯
D.	◯	◯	◯	◯	◯	◯
E.	◯	◯	◯	◯	◯	◯
F.	◯	◯	◯	◯	◯	◯

Please use this image to answer the questions below.

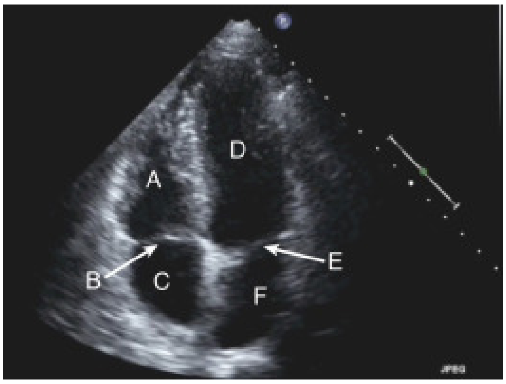

Name the cardiac view depicted above.

◯ Parasternal Long
◯ Parasternal Short
◯ Apical 4-chamber
◯ Subcostal
◯ Transesophageal
◯ None of the above

Please name each of the structures labeled in the image above.

	Right atrium	Right ventricle	Left atrium	Left ventricle	Mitral Valve	Tricuspid valve
A.	◯	◯	◯	◯	◯	◯
B.	◯	◯	◯	◯	◯	◯
C.	◯	◯	◯	◯	◯	◯
D.	◯	◯	◯	◯	◯	◯
E.	◯	◯	◯	◯	◯	◯
F.	◯	◯	◯	◯	◯	◯

Please use this image to answer the questions below.

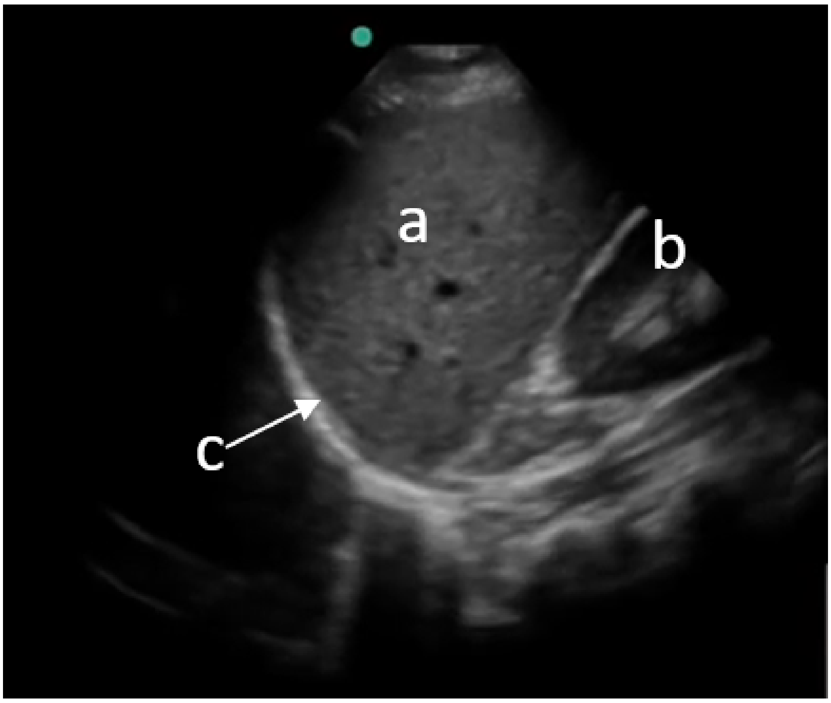

Please name each of the structures labeled in the image above.

	A. Liver	B. Diaphragm	C. Kidney
A.	◯	◯	◯
B.	◯	◯	◯
C.	◯	◯	◯

The space between the liver and kidney where fluid may accumulate is referred to as the pouch of Douglas.

◯ True
◯ False

Use the following probe orientations to answer the questions below [The dot indicates the probe marker].

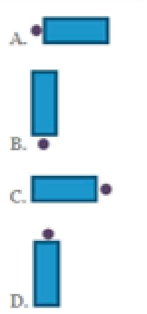

	A.	B.	C.	D.
A longitudinal view of the IVC.	○	○	○	○
Lung ultrasound.	○	○	○	○
Transverse approach for central line access.	○	○	○	○
